# Multi-scale fractal Fourier Ptychographic microscopy to assess the dose-dependent impact of copper pollution on living diatoms

**DOI:** 10.1038/s41598-024-52184-3

**Published:** 2024-04-10

**Authors:** Vittorio Bianco, Lisa Miccio, Daniele Pirone, Elena Cavalletti, Jaromir Behal, Pasquale Memmolo, Angela Sardo, Pietro Ferraro

**Affiliations:** 1 CNR-ISASI, Institute of Applied Sciences and Intelligent Systems “E. Caianiello”, Via Campi Flegrei 34, 80078 Pozzuoli, Naples, Italy; 2 Marine Biotechnology Department, Stazione Zoologica Anton Dohrn, Villa Comunale, 80121 Naples, Italy; 3 Department of Chemical, Materials and Production Engineering, University of Naples Federico II, Piazzale Tecchio 80, 80125 Naples, Italy

**Keywords:** Environmental sciences, Optical sensors, Computational science, Environmental biotechnology

## Abstract

Accumulation of bioavailable heavy metals in aquatic environment poses a serious threat to marine communities and human health due to possible trophic transfers through the food chain of toxic, non-degradable, exogenous pollutants. Copper (Cu) is one of the most spread heavy metals in water, and can severely affect primary producers at high doses. Here we show a novel imaging test to assay the dose-dependent effects of Cu on live microalgae identifying stress conditions when they are still capable of sustaining a positive growth. The method relies on Fourier Ptychographic Microscopy (FPM), capable to image large field of view in label-free phase-contrast mode attaining submicron lateral resolution. We uniquely combine FPM with a new multi-scale analysis method based on fractal geometry. The system is able to provide ensemble measurements of thousands of diatoms in the liquid sample simultaneously, while ensuring at same time single-cell imaging and analysis for each diatom. Through new image descriptors, we demonstrate that fractal analysis is suitable for handling the complexity and informative power of such multiscale FPM modality. We successfully tested this new approach by measuring how different concentrations of Cu impact on *Skeletonema pseudocostatum* diatom populations isolated from the Sarno River mouth.

## Introduction

Heavy Metals (HMs) in water and sediments could derive from natural sources, such as metal-bearing rocks and volcanic eruptions, since they naturally occur within the Earth Crust. However, high HM concentrations are usually an indicator of anthropogenic impacts on aquatic environments, and are a common feature of industrialized and highly urbanized areas^1^. Accumulation of HMs is a serious issue for aquatic ecosystems because of their persistence (they are non-degradable elements) and the toxicity of their bioavailable fraction (that is usually the soluble one) for living organisms. Furthermore, trophic transfer of HMs through the food-chain may also increase biomagnification risks, and cause serious damages to human health^1,2^. HM pollution assessments can be carried out relying on chromatography, spectroscopy or electrochemistry. Traditional devices based on chromatography and spectroscopy present very high performance in terms of sensitivity, specificity and accuracy but require expert and skilled personnel and many sample pre-processing steps. Furthermore, such lab-based methodologies, e.g. high-performance liquid chromatography (HPLC)^3^, atomic absorption spectrometry^4^, colorimetry^5^, and fluorescence spectroscopy^6^ are expensive and not-rapid in terms of response time so that they are impractical for routine in-situ monitoring. Recently, great effort has been spent in developing tools for HM detection at Lab-On-Chip scale in compliance with the regulation rules that are continuously updated in terms of maximum concentration levels^7^. Portable devices have been realized based on Surface-Enhanced Raman Scattering spectroscopy (SERS)^8^, electrochemistry^9^, integrable microfluidics^10^, and handheld colorimetry devices^11^. All of them detect and quantify HMs with the common drawback of not evaluating the effect on the native species. Another drawback is the massive usage of chemical signal transducers as dyes or nanoparticles. Placido and co-authors^12^ trace the way toward a more green-oriented approach where biochar produced by microalgae biofuel is transformed into carbon dots and applied as HM ion sensors in aqueous systems. However, the sole detection of HMs and their concentration in a certain habitat is not sufficient to understand the impact these pollutants might have on aquatic ecosystems. Only a fraction of the HMs present in water can be bioavailable and, thus, internalized by living organisms: for example, copper (Cu) can be complexed to organic ligands, limiting its toxic effects^13^. Microalgal response to HMs can also vary among species^14^, and this is mostly due to different strategies to minimize HM detrimental effects. Moreover, some HMs at low concentrations provoke only negligible toxicological effects on microalgal cells. Thus, it is of growing interest to study the HM pollution from a functional perspective, i.e. by assaying the effects of HMs on microbial organisms in relation to their concentration. A new paradigm has gained interest recently where functional biomarkers are employed to assess the eco-toxicological impact of pollutants and especially the effects of HMs on the environment^15^. Diatoms have shown good sensitivity in terms of biological responses to the magnitude and exposure of environmental contaminants^16^. It has been proved that HM toxicants might inhibit algal growth rates and photosynthesis thus behaving as bioindicators^17^.

Here we assess the effect of HM pollutants on microalgae by adopting a multi-scale microscopy analysis based on Fourier Ptychographic Microscopy (FPM). Moreover, we introduce new descriptors of the FPM image as a whole, which rely on fractal geometry, to evaluate the impact of different concentrations of Cu on a diatom population. As a test case, we investigate the diatom *Skeletonema pseudocostatum*, isolated from the Italian Sarno River mouth. Among HMs, Cu is toxic and can induce malformations to a wide range of freshwater and marine organisms^18,19^. In aquatic environments, HM contamination may also affect primary producers such as microalgal cells, affecting growth and causing impairments in their shape and organelles as a consequence of HM adsorption and/or internalization. At low (e.g. nanomolar) concentrations, Cu is an essential metal for algal growth, metabolism and enzyme activities^20^. However, at increasing concentrations, Cu disturbs the integrity and the permeability of microalgal cell membranes^21,22^. Inhibition or limitation of algal growth rates is an issue of special importance, since microalgae play a key role in carbon fixation in the oceans^23^ and serve as a natural food source for zooplankton^24^ and filter feeders^25^. The use of diatom species^26^ as bioindicators is particularly advantageous since they are the most widespread class in almost all freshwater and marine environments. The genus *Skeletonema* is widely distributed in the Gulf of Naples^27^. The dose-dependent response of this species to different Cu concentrations paves the way to its exploitation as biosensor for detection of phenomena of Cu pollution in marine environments. In this work, we rely on a FPM system^28–33^ to image and infer information from diatoms exposed to various Cu doses. FPM belongs to the wide category of label-free Quantitative Phase Imaging (QPI) methods^34,35^. QPI does not require dyes or markers and uses refractive index differences of samples probed in through transmission as an endogenous contrast agent. Hence, QPI minimizes sample preparation, is non-invasive and gives access to the specimen morphometric information. Independently on the contrast mechanism exploited, any imaging system, ranging from benchtop commercial devices to custom optical setups, has to trade-off imaging Field of View (FoV), depth of focus and spatial resolution. Offering quantitative label-free imaging with large space-bandwidth product^28^ is the holy grail to analyse a statistically relevant number of algal bio-probes without sacrificing resolution. According to a synthetic aperture principle, FPM illuminates the sample from multiple directions and acquires a set of bright-field and dark-field intensity images. Each of them carries a different set of spatial frequencies of the illuminated object, from the DC component to the ones representing the finest details^28–33^. An iterative image synthesis and phase retrieval algorithm allows phase-contrast mapping of the samples beyond the resolution limit of the optical system, with wide FoV and large depth of focus. Due to its features, FPM can be thought as a multi-scale microscopy method that offers both the ensemble view of the sample of liquid containing the algal population and an insightful high-resolution characterization of each single diatom element. In the case of the *Skeletonema pseudocostatum*, the developed FPM system can image thousands of diatoms in one single FoV with single-cell level spatial resolution, of the order of 0.5 µm. In order to fully exploit these features, for the first time we propose fractal geometry as a multi-scale analysis tool of the FPM maps. Fractal geometry outperforms Euclidean approaches in describing the complexity of nature and is widely exploited to model and analyze, at tunable scale, the roughness and topography of natural surfaces, plant leaves and crop fields, landscapes and coastlines, and biological elements, e.g. tissues^36–39^. In this framework, the concepts of fractal dimension and lacunarity emerged as ground-breaking and can be used to infer information on the way an object, or groups of them, fill spaces or volumes^40,41^. The fractal dimension can take non integer values and does not coincide with the Euclidean dimension for a large percentage of natural objects and living species. Similarly, lacunarity measures the distribution of gaps and their asymmetries in an object at various scales. Recently, we used QPI and fractal geometry to define all-optical footprints at the single particle level to identify microplastics among a wide heterogeneity of microalgae species^41^.

**Figure 1:**
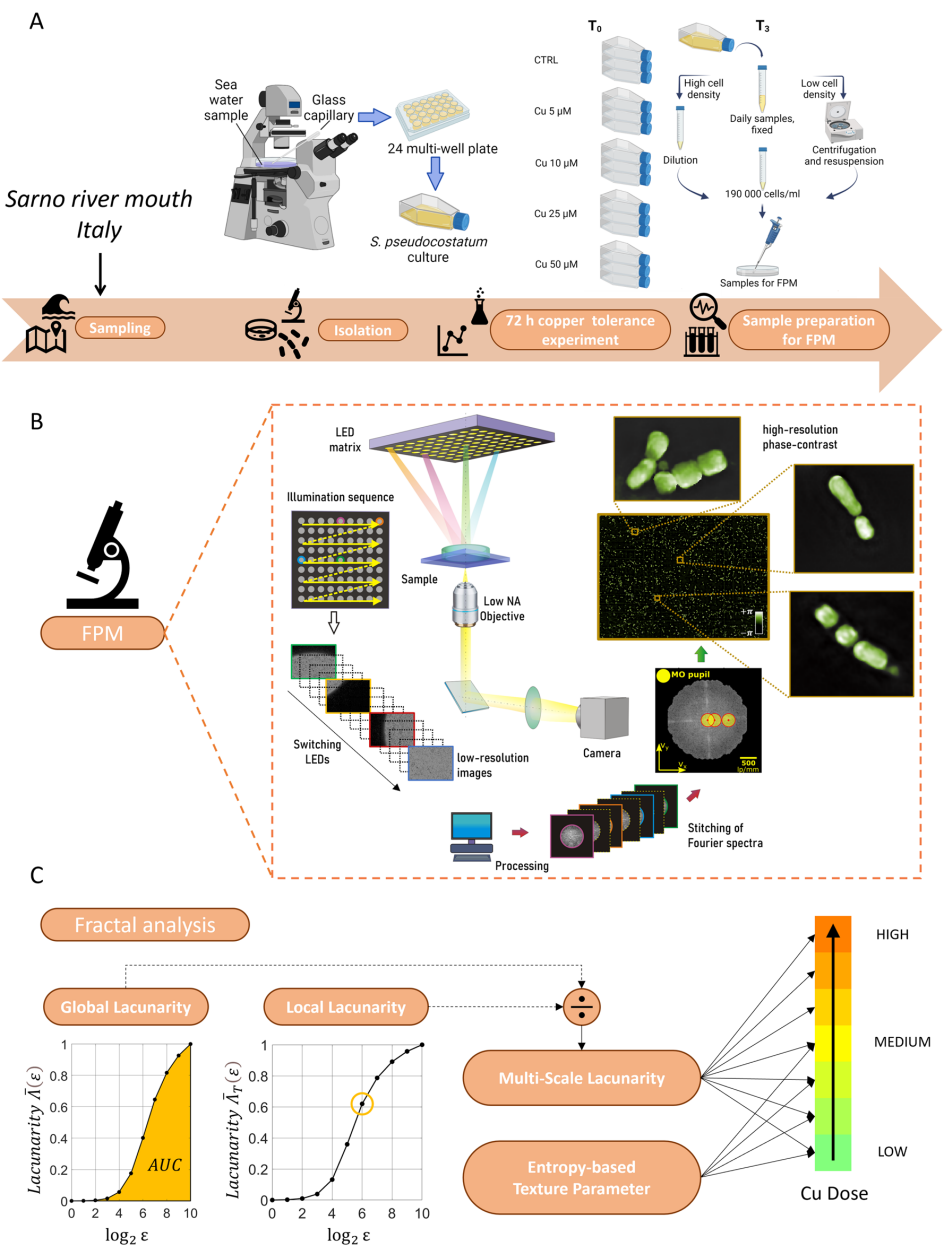
Analysis protocol to use diatoms as bio-probes of Cu doses in water. (**A**). Sampling and preparation pipeline to create the sensor calibration curves. (**B**). Sketch of the multi-scale FPM imaging device. The output is the high-resolution phase-contrast map that we use for downstream fractal analysis. (**C**). Multi-scale analysis of FPM maps of marine diatoms based on fractal geometry. In the inset we report example plots of global and local lacunarity parameters. As a result of the analysis, each water sample is associable to one interval of Cu doses out of seven. Figure 1A: Created with BioRender.com.

Here we define the Multi-Scale Lacunarity (MSL) descriptor and apply it to FPM phase-contrast images to characterize the concentration of Cu the algal bio-probes are exposed to, Fig. 1. The joint action of the multi-scale FPM imaging and the MSL characterizes, as a whole, the sample of liquid containing a multitude of marine diatoms and, at once, the contribution of each diatom at single cell level, spanning from the millimeter range to the micron scale in one snapshot. Following this approach, we turn the FPM microscope into a sensor that can monitor the concentration of Cu through the effects it has on the bio-sentinels. In particular, we prove that the FPM imaging combined to an analytical tool based on the fractal geometry is able to detect seven possible Cu dose intervals spanning both low and high contamination levels. We believe that the attained results open a route towards the creation of a new sensor for a fast and automatic quantification of the aquatic pollution level in terms of its effects on the microbial populations.

## Materials and methods

### Sampling, preparation and exposure of diatom probes to Cu

*Skeletonema pseudocostatum* was isolated on June 2020 from water samples collected from the Sarno River mouth (40.7291N, 14.4698E) by hand pipetting, correctly identified by PCR amplification and sequencing of 18S and 28S genes (Fig. 1A), and treated as described in Supporting Information. *Skeletonema pseudocostatum* was then exposed to scalar Cu concentrations to obtain the following final concentrations: 0 (e.g. control conditions), 5 µM, 10 µM, 15 µM, 25 µM, 35 µM and 50 µM (see Supporting Information). Concentration was daily assessed at different time intervals (0, 24, 48 and 72 h), hereafter referred to as T_0_, T_1_, T_2_, T_3_, respectively. Experimental samples were all acquired as described in Bianco et al.^30^ at the same cell density (around 190,000 cells/ml), obtained by appropriate dilution in f/2 or centrifugation (4 °C, 2700 g, 10 min, Thermo Scientific SL16R) and resuspension of the cells in the desired volume. For each acquisition, 900 µl of lugol-fixed sample were placed in a Petri dish (glass bottom WillCo-dish® GWST-5040, 40 mm aperture diameter, 7 mm height) to form a very thin film of liquid to obtain images of in-focus diatoms. 3 FoVs were acquired for each sample. In particular, we focused on the exposure times T_2_ and T_3_, since the exposure times T_0_ and T_1_ are expected to be too short to observe significant differences among diatoms subjected to different Cu doses. As regards the exposure time T_2_, we recorded PCMs for each Cu concentration under test. The same procedure was repeated in the T_3_ case, with the sole exception of the highest concentration tested (50 μM), that caused a consistent reduction of cell density and was considerably higher than half maximal effective concentration.

### FPM principle

All the FPM acquisitions consist of illuminating the object with plane waves distinct in angular inclinations while simultaneously capturing the corresponding intensity images. This procedure can be clearly understood considering that fine structures of the sample transmit through the low-pass filtering imaging system when the sample is probed with oblique illumination, Fig. S1. Sequential-illumination FPM measurements of a thin object o(**r**) with a transversal vector **r** = (x,y) are often accomplished with an LED array placed far enough from the sample; thus, the probing can be considered a locally-coherent plane-wave illumination of a central wavelength λ. Consequently, each of the N used LEDs contains the spatial carrier frequency ${{\varvec{\nu}}}_{{\varvec{n}}}=({\nu }_{xn},{\nu }_{yn})$, n = 1,…,N. The light behind the sample is straightforwardly considered a product of the sample and the oblique illumination optical fields as $o\left( {\varvec{r}} \right)exp\left( {i2\pi {\varvec{\nu}}_{{\varvec{n}}} \cdot {\varvec{r}}} \right)$, which generates a shift of the object’s Fourier spectrum O(${{\varvec{v}}-{\varvec{v}}}_{{\varvec{n}}}$). Here $O({\varvec{\nu}})=FT\{o({\varvec{r}})\}$ represents the original-sample Fourier spectrum with FT{} being the 2D Fourier transform operator. The optical field subsequently passes the imaging system serving as a low-pass filter of a pupil function $P({\varvec{\nu}})$. The truncating function $P({\varvec{\nu}})$ is commonly considered a circle of radius NA_MO_/λ, where NA_MO_ is numerical aperture of the used microscope objective (MO); thus, the field arising at the pupil of the MO is expressed as the product of $O({\varvec{\nu}}-{{\varvec{v}}}_{{\varvec{n}}})$ and $P({\varvec{\nu}})$. The low-resolution intensity image created in the detector plane is then calculated as ${I}_{n}({\varvec{r}})={|IFT\{O({\varvec{\nu}}-{{\varvec{v}}}_{{\varvec{n}}})P({\varvec{\nu}})\}|}^{2}$, where IFT{} represents the 2D inverse Fourier transform operator.

The primary motivation of the complex-amplitude retrieval process is to jointly estimate the object function $O({\varvec{\nu}})$ and the pupil function $P({\varvec{\nu}})$, the latter containing the optical system aberrations that may distort the image quality^42^. The whole sequence of N low-resolution intensity snapshots ${I}_{n}({\varvec{r}})$, n = 1,…,N, serves as the input of the retrieval algorithm, where the up-sampled version of the central low-resolution bright-field image acts as an initial guess of the object, and the pupil function is initialized as a circle of radius NA_MO_/λ and the constant phase. All the captured images are addressed sequentially from n = 1 to n = N and considered in this order, with both the pupil function and sample spectrum being updated in each of the j-th loop (j = 1,…,J)^42^.

In the j-th iteration of the retrieval algorithm, functions ${P}^{(j)}({\varvec{\nu}})$ and ${O}^{(j)}({\varvec{\nu}})$ are created using their estimates obtained in the previous (j−1)-th loop. The output field of the object illuminated by the n-th LED in the pupil plane of the used microscope objective is calculated as ${\varphi }^{(j-1)}({\varvec{\nu}})={O}^{(j-1)}({\varvec{\nu}}-{{\varvec{v}}}_{{\varvec{n}}}){P}^{(j-1)}({\varvec{\nu}})$, and the corresponding low-resolution complex-amplitude image in the detector plane is simulated by its inverse Fourier transform as ${\Phi }^{(j-1)}({\varvec{r}})=IFT({\varphi }^{(j-1)}({\varvec{\nu}}))$. Subsequently, based on the Gerchberg-Saxton-Fienup retrieval approach, the amplitude of such complex distribution is replaced by the square root of the measured low-resolution intensity image; thus, ${\Phi {\prime}}^{(j-1)}({\varvec{r}})={({I}_{n}({\varvec{r}}))}^{1/2}{\Phi }^{(j-1)}({\varvec{r}})/|{\Phi }^{(j-1)}({\varvec{r}})|$ and the updated pupil plane distribution are calculated according to ${\varphi {\prime}}^{(j-1)}({\varvec{\nu}})=FT({\Phi {\prime}}^{(j-1)}({\varvec{r}}))$. Following the gradient-descent principle, both estimates of ${O}^{(j-1)}({\varvec{\nu}})$ and ${P}^{(j-1)}({\varvec{\nu}})$ are further updated based on previous calculations as

1 $${O}^{(j)}({\varvec{\nu}})={O}^{(j-1)}({\varvec{\nu}})+{G}^{(j-1)}({\varphi \mathrm{^{\prime}}}^{(j-1)}({\varvec{\nu}}+{{\varvec{\nu}}}_{{\varvec{n}}})-{\varphi }^{(j-1)}({\varvec{\nu}}+{{\varvec{\nu}}}_{{\varvec{n}}}))$$

and

2 $${P}^{(j)}({\varvec{\nu}})={P}^{(j-1)}({\varvec{\nu}})+{H}^{(j-1)}({\varphi \mathrm{^{\prime}}}^{(j-1)}({\varvec{\nu}})-{\varphi }^{(j-1)}({\varvec{\nu}})),$$

respectively. The functions ${G}^{(j-1)}$ and ${H}^{(j-1)}$ vary during each iteration due to their dependency on the estimated pupil function, object’s Fourier spectrum, and additional parameters as

3 $${G}^{\left(j-1\right)}\left({\varvec{\nu}}\right)= \frac{\left|{P}^{\left(j-1\right)}({\varvec{\nu}}+{{\varvec{\nu}}}_{{\varvec{n}}})\right|{\left({P}^{\left(j-1\right)}({\varvec{\nu}}+{{\varvec{\nu}}}_{{\varvec{n}}})\right)}^{*}}{{\text{max}}\left(\left|{P}^{\left(j-1\right)}({\varvec{\nu}})\right|\right)\left({\left|{P}^{\left(j-1\right)}({\varvec{\nu}}+{{\varvec{\nu}}}_{{\varvec{n}}})\right|}^{2}+{\delta }_{1}\right)},$$

4 $${H}^{\left(j-1\right)}\left({\varvec{\nu}}\right)= \frac{\left|{O}^{\left(j-1\right)}({\varvec{\nu}}-{{\varvec{\nu}}}_{{\varvec{n}}})\right|{\left({O}^{\left(j-1\right)}({\varvec{\nu}}-{{\varvec{\nu}}}_{{\varvec{n}}})\right)}^{*}}{{\text{max}}\left(\left|{O}^{\left(j-1\right)}({\varvec{\nu}})\right|\right)\left({\left|{O}^{\left(j-1\right)}({\varvec{\nu}}-{{\varvec{\nu}}}_{{\varvec{n}}})\right|}^{2}+{\delta }_{2}\right)},$$

where * represents a complex conjugation, ${\text{max}}(\Sigma )$ is maximum of $\Sigma$, and ${\delta }_{1}$, ${\delta }_{2}$ are regularization constants ensuring numerical stability^43^.

Simultaneous retrieval of the sample spectrum and the pupil function enhance the accuracy of the complex-amplitude recovering process. These updates are repeated until all n = 1,…,N captured low-resolution intensity images are used, which can be considered a single complete iteration that recovers the high-resolution spectrum. This single complete iteration is repeated J times to improve convergence, and in turn to get the steady-state pupil function and the sample spectrum estimates ${P}^{(J)}({\varvec{\nu}})$ and ${O}^{(J)}({\varvec{\nu}})$. Finally, the obtained high-resolution spectrum ${O}^{\left(J\right)}\left({\varvec{\nu}}\right)$ is inverse Fourier transformed to obtain the high-resolution complex-amplitude image of the investigated specimen ${o}^{\left(J\right)}\left({\varvec{r}}\right)$. More details about the used algorithm are included in the reference^43^.

## Results

**Figure 2:**
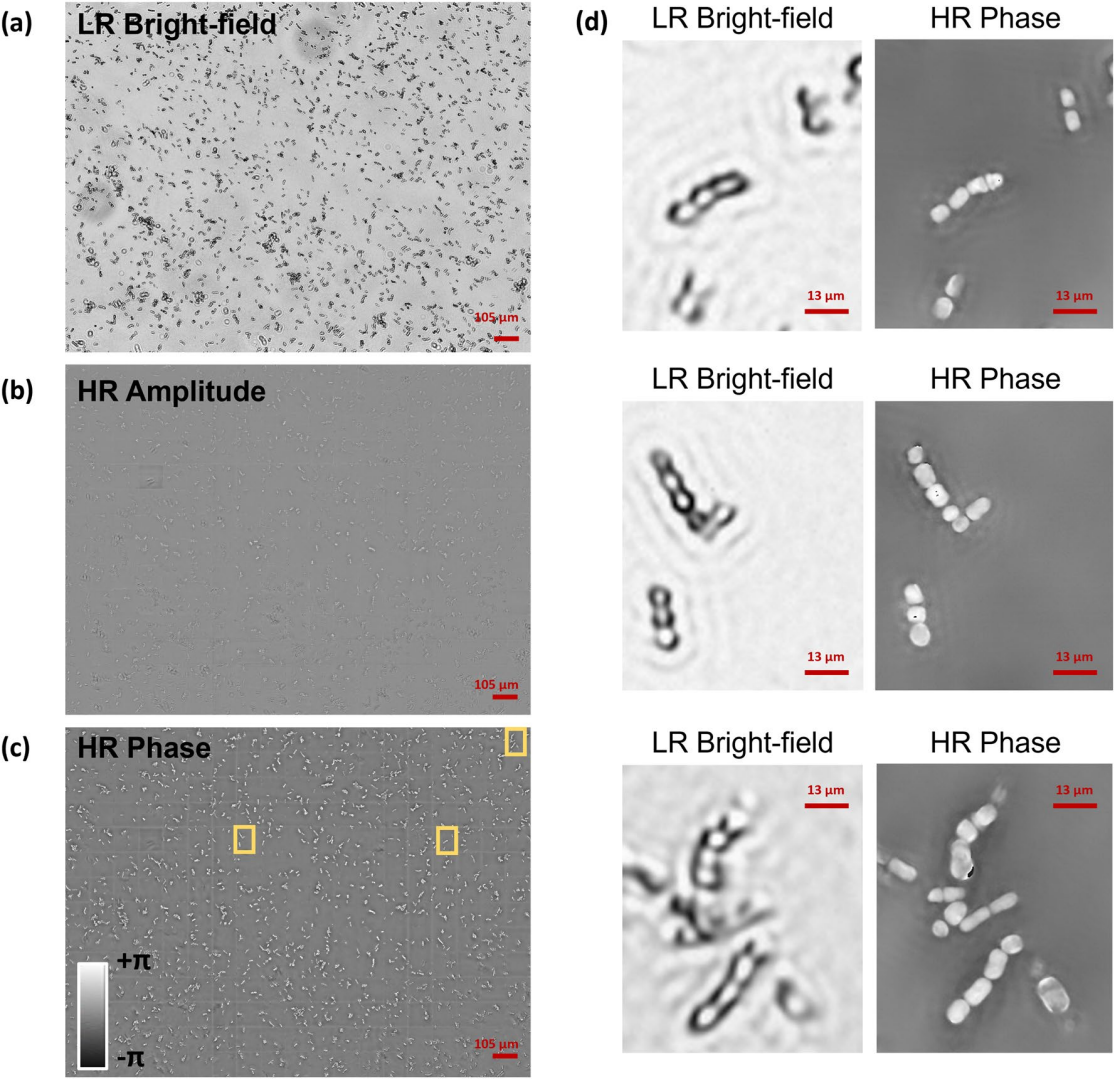
FPM processing results. (**a**) Full FoV LR bright-field intensity, central LED. (**b**) Full FoV reconstructed HR amplitude. (**c**) Full FoV reconstructed HR phase-contrast map. (**d**) Zoom-in details corresponding to the areas marked by yellow boxes in (**c**).

**Figure 3:**
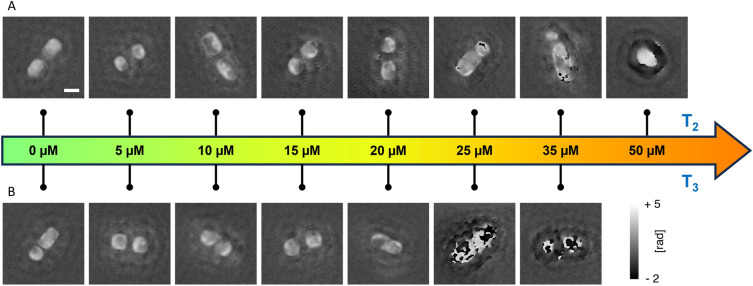
details of FPM phase-contrast maps obtained from experiments at different Cu doses and exposure times.

Samples were analysed using a custom FPM inverted microscope, sketched in Fig. 1B and described in the Supporting Information (Fig. S1). Here we used FPM to image in the same large FoV hundreds of diatoms, used as bio-probes after being exposed to controlled Cu doses. Indeed, the low 4×*g* permits accessing a wide 3.3 mm^2^ FoV, while the FPM synthetic aperture principle allows super-resolved quantitative phase-contrast imaging, with 0.5 μm lateral resolution. This unique feature of FPM allows imaging in the same FoV large numbers of diatoms on a millimetric scale while preserving the micron-scale characteristics of single diatom elements (e.g., see the insets in Fig. 1B). An example of FPM processing is reported in Fig. 2 for one of the control acquisitions. We report (a) the full FoV of one of the 177 captured images that corresponds to the Low Resolution (LR) bright-field intensity obtained by switching on the central LED, (b) the full FoV reconstructed High Resolution (HR) amplitude and (c) the full FoV reconstructed HR phase-contrast map. The HR phase-contrast map is the one we use for the further fractal analysis. In particular, in Fig. 2d we show some zoom-in details corresponding to the areas marked by yellow boxes in Fig. 2c, where the effect of super-resolution processing and phase retrieval is apparent from the comparison between the LR bright-field and the HR phase-contrast. Figure 3 shows one characteristic example of diatoms imaged by FPM for each exposure time and Cu dose. The phase-contrast maps at the single diatom level are shown. In some cases, especially for the highest Cu doses, diatoms experience morphological variations and/or cytoplasm extrusion after membrane lysis. In these cases, the variation of the phase contrast is made noticeable by observing the phase-contrast map. However, there are cases (especially for the sub-lethal doses) where the morphological variations are not so evident for the single diatom and membrane lysis did not occur.

In order to fully exploit the multi-scale property of FPM, it is convenient to look for the analysis methodology in the framework of multi-scale math categories. So far, fractal geometry has been demonstrated the most powerful instrument able to provide an insight of nature deeper than its Euclidean counterpart. Indeed, its main credit lies in the ability of breaking the mold of the classical geometry that frames the topological dimension of an object in the realm of integer numbers, thus pushing towards a fascinating non-integer vision of reality^44^. To accomplish this goal, we inspected the samples by a multi-scale fractal analysis that sinks its roots on the box-counting concept. Among the possible fractal parameters that could be considered, here we focused on lacunarity^44–46^. Lacunarity can be thought as the distribution of hole sizes inside a single object^41^ or within a group of objects imaged in the same FoV, e.g. a tissue or confluent cell layers^46^. Lacunarity is expected to provide a reliable solution to the problem we are tackling, since it is a quantitative descriptor very sensitive to the tiny dissimilarities between the PCMs of the bio-sentinels exposed to different Cu doses. We introduced two lacunarity-based parameters (Fig. 1C), here defined as the global lacunarity (GL) and the local lacunarity (LL), described in detail in the Supporting Information section. The global lacunarity GL is an ensemble descriptor of the hole sizes distribution computed at different scales and therefore it considers the global context of the PCM, i.e. both the diatom probes and the background medium. Instead, the local lacunarity LL measures the hole sizes distribution at a fixed scale, properly selected here in order to consider only the local interplay between the bio-probes and the medium at the single-diatom level. The following calibration experiments and the proposed test are based on the Multi-Scale Lacunarity (MSL) parameter, defined here as the ratio between the global and the local lacunarities and used to measure the Cu concentration the imaged diatoms were exposed to.

### Calibration

**Figure 4:**
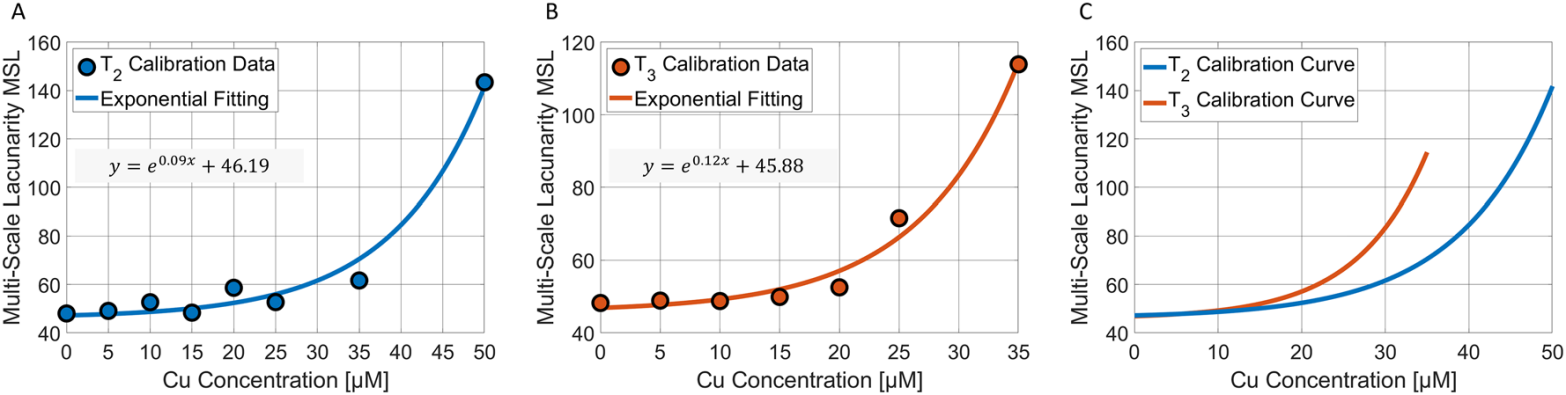
Calibration of the system for detecting the Cu dose. A,B Multi-scale lacunarity of the calibration data (dots) about the FPM-PCMs with diatoms recorded respectively after T_2_ and T_3_ exposure times at different Cu doses. The equations of the best exponential fittings (solid lines) are reported. C Comparison between the T_2_ and T_3_ calibration curves.

We employed the described pipeline of FPM and multi-scale fractal analysis to record and characterize liquid samples made of diatoms exposed to different Cu doses for both the exposure times T_2_ and T_3_. The two exposure times have been treated separately, as shown in Fig. 4A,B. For each Cu dose, we measured the MSL from the PCMs and exploited it to calibrate the system. As desired, and also expected on the basis of the previous considerations, the corresponding dots reported in Fig. 4A,B follow monotonic trends with the Cu dose. Therefore, we fitted them by exponential curves (solid lines), used here as reference calibration curves. The comparison between the T_2_ and T_3_ calibration curves in Fig. 4C is remarkable. Indeed, the two curves start from the same MSL value at 0 μM (control sample), despite they were obtained independently of each other from different experimental sets and acquired in different days in order to avoid any experimental bias.

Moreover, they have comparable MSL values at low doses, and strongly diverge at high doses. Measuring the same starting point underlines the robustness and repeatability of this strategy and serves as a negative control for the sensor, since the diatoms in the control images were not exposed to any Cu dose, therefore no difference between the T_2_ and T_3_ cases should have emerged. Instead, the divergence at high doses is a first validation of this system on the basis of biological considerations. In fact, because of the longer exposure time, the T_3_ curve grows faster with the Cu dose than the T_2_ curve, since diatoms are more impacted by the toxic effects due to the longer Cu internalization, and therefore they start exhibiting discrepancies with respect to the control at lower Cu doses. Furthermore, the steepest trend of the T_3_ curve leads to a greater sensitivity of the proposed sensor in detecting the correct Cu dose intervals with respect to the T_2_ curve, as larger MSL differences can be measured in correspondence of the same Cu dose interval. Hence the curves shown in Fig. 4 put in evidence the capability of the system and the overall measurement and analysis protocol of sensing this difference of impact on the bio-sentinels.

### Testing and sensitivity enhancement

**Figure 5:**
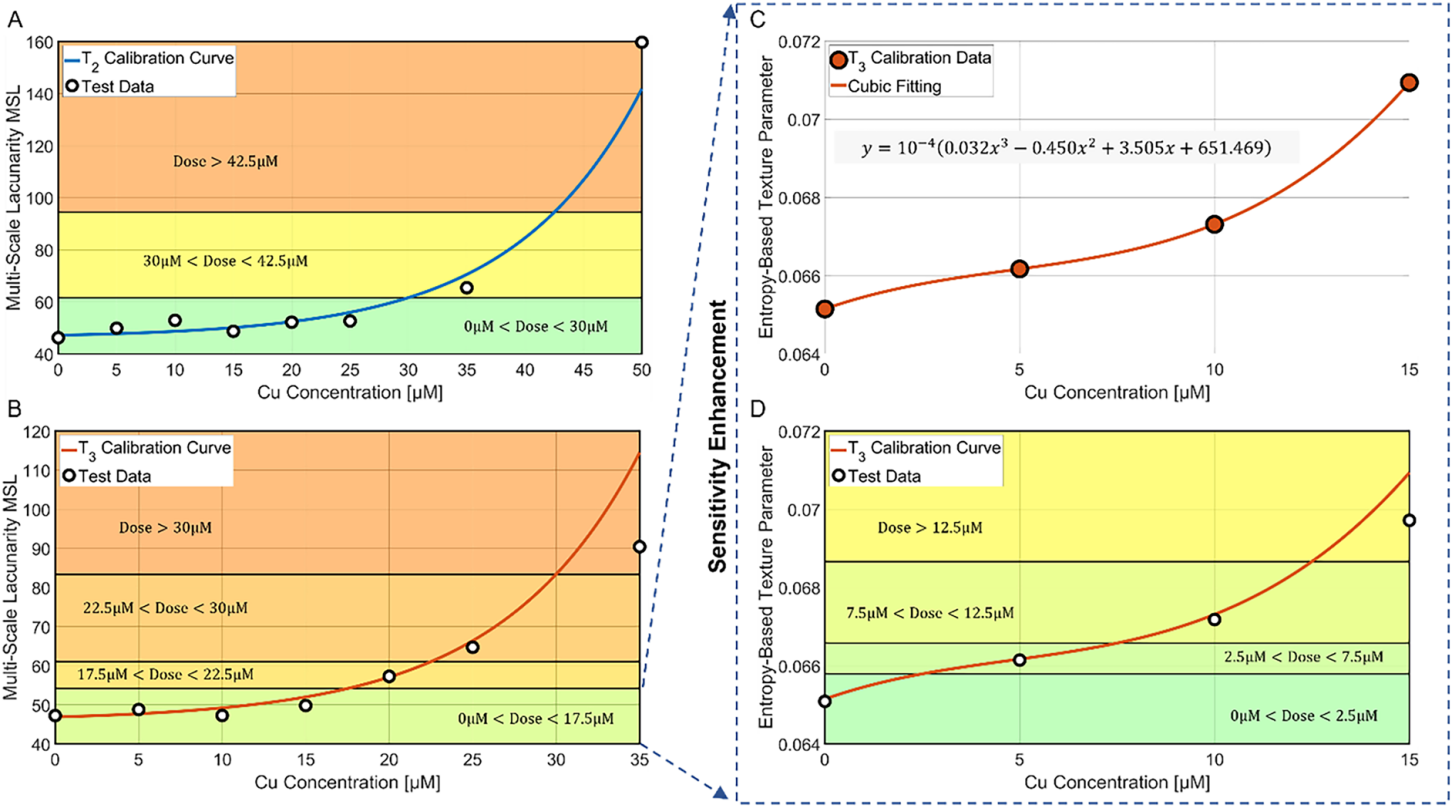
Testing the system for detecting the Cu dose. A,B MSL calibration curves (solid lines) with overlapped the test data (dots) about the FPM-PCMs with diatoms recorded respectively after T_2_ and T_3_ exposure times at different Cu doses. The detectable Cu ranges are highlighted. C Entropy-based texture parameter of the calibration data (dots) about the FPM-PCMs with diatoms recorded after T_3_ exposure time at different Cu low-doses. The equation of the best cubic fitting (solid lines) is reported. D Entropy-based calibration curve (solid line) with overlapped the test data (dots) about the FPM-PCMs with diatoms recorded after T_3_ exposure time at different Cu low-doses. The detectable Cu ranges with higher sensitivity at the low doses are highlighted.

In order to test the ability of the calibrated system in measuring a certain Cu dose on the basis of the MSL, in Fig. 5A,B we report as black dots the MSL values obtained from PCMs not considered during the calibration step, overlapped to the T_2_ and T_3_ calibration curves, respectively. As for the exposure time T_2_ (Fig. 5A), due to the lower slope of the calibration curve, only three Cu dose intervals can be detected, i.e. [0,30] μM, [30,42.5] μM, and [42.5,∞) μM. Instead, the greater slope of the T_3_ case displayed in Fig. 5B allows revealing four Cu dose intervals, i.e. [0,17.5] μM, [17.5,22.5] μM, [22.5,30] μM, and [30,∞) μM. On the basis of the reported analysis, we conclude that the exposure time T_3_ is the optimal solution to determine the unknown Cu doses due to the greater sensitivity of the system. This is particularly important for discriminating the Cu doses in the lowest concentrations range, where the current biological procedures are not able to catch very small changes among the different Cu effects. Moreover, the exposure time T_3_ also allows an enhancement of the system sensitivity in finding the lowest Cu doses within the [0,17.5] μM interval. Toward this scope, we characterized each PCM through a more conventional entropy-based texture parameter, i.e. the range GLCM entropy ∆s described in the Supporting Information and shown in Fig. S4O. Entropy is well-known as a metric of the disorder of a system. Hence, entropy is expected to grow with the Cu dose because, as discussed in the previous section, the sample goes from a clean medium with embedded healthy elements to a dirtier medium with elements undergoing membrane lysis that provokes cytoplasm leaks, i.e. a situation with a higher disorder level. This hypothesis is verified in Fig. 5C, where the entropy-based texture parameter corresponding to the calibration doses follow again a monotonic trend, but with more marked differences among them in the [0,15] μM interval. Therefore, we used a cubic fitting to create the low-dose calibration curve (solid line in Fig. 5C). Finally, as shown by the test PCMs (black dots in Fig. 5D) overlapped to the entropy-based calibration curve, three Cu sub-intervals can be found in correspondence to the lowest doses range, i.e. [0,2.5] μM, [2.5,7.5] μM, [7.5,12.5] μM, and [12.5,17.5] μM. In summary, as reported in Table 1, the proposed test for detecting the impact of different Cu concentrations is made of two steps. The former exploits the fractal geometry to compute the MSL, which value yields four possible Cu dose intervals, i.e. [0,17.5] μM, [17.5,22.5] μM, [22.5,30] μM, and [30,∞) μM. The latter is performed only in the case the MSL measured at the end of the first step is below the 17.5 μM value. In such a case, the system sensitivity is enhanced by computing a PCM entropy-based texture parameter, thus providing four further Cu low-dose sub-intervals, i.e. [0,2.5] μM, [2.5,7.5] μM, [7.5,12.5] μM, and [12.5,17.5] μM. It is worth noting that, as reported in Fig. S4O, even if the ${T}_{3}$ curve has a monotonic trend, the sole entropy-based texture parameter cannot replace the MSL in the first step of the detection system since the basic congruence between the T_2_ and T_3_ data discussed in Fig. 4C is missing when the Cu dose is higher than 15 μM. In the Supporting Information and Fig. S4, other conventional features have been tested, but none of them shows the congruence property exhibited by the fractal analysis (see Fig. S4F,H,K,L). Some features do not show a monotonic trend even in the T_3_ case (see Fig. S4A-E,G,I,J,M,N). This congruence property is instead pivotal as it gives robustness to the proposed Cu detection system, since the output of the fractal analysis is able to replicate what is expected from the biological point of view, i.e. it is able to provide a parameter that follows a monotonic trend in both the T_2_ and T_3_ cases but with different growth rates due to the different exposure times, while starting from the same value corresponding to the negative control.

**Table 1:**
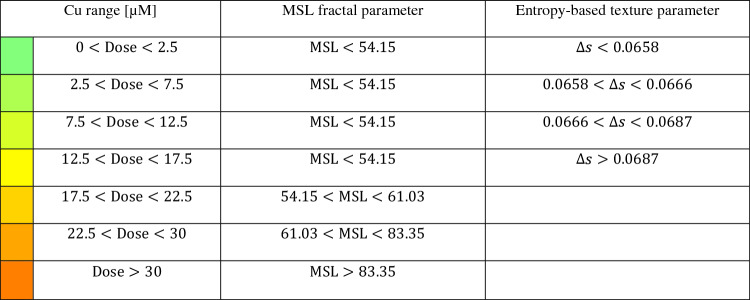
Thresholds of the MLS Fractal Parameter and Entropy-based Texture Parameter for detecting different Cu Ranges inside a water sample.

### Fractal-FPM timing analysis

The time needed for detecting the Cu dose can be considered as the sum of three terms.

- The first term is related to the FPM recording time. In fact, 177 low-resolution images are acquired in about 4 min. However, it has been demonstrated that the number of employed LEDs can be reduced without losing resolution by employing coded illumination schemes.
- The second term is the computational time requested by the phase retrieval algorithm for obtaining a high-resolution FPM map from the recorded 177 low-resolution intensities. We have demonstrated in a previous work that, using deep learning, the phase retrieval can be greatly speeded up, thus taking about 1 min for reconstructing the entire 7000 × 9500 (i.e., 3.3 mm^2^) FoV^33^.
- The third term is the computational time requested by the fractal analysis for computing the global lacunarity, which is about 5–6 min for each 7000 × 9500 (i.e., 3.3 mm^2^) FoV. However, due to the structure of the fractal analysis algorithm, parallel computing along with the use of GPUs could significantly speed up this process.

## Conclusions and discussion

High doses of the Cu bioavailable fraction can play a negative role in the morphology, growth, survival rate, and reproduction of primary producers. Sensors have been proposed to detect and quantify the concentration of HMs in water. SEM has been already employed in experiments performed on *Skeletonema spp*. exposed to metals, but it was mainly employed to study and assess the distribution of metal nanoparticles in the culture media rather than to observed eventual impairments in the algal morphology^47^. Moreover, metal-treated cultures can be characterised by cell aggregations and metal sorption on the cell wall which make difficult a cell- by cell observation at SEM or TEM microscopes^48,49^. This problem could be partially overcome by diluting samples before the observation, but this strategy further limits the number of cells which can be observed, rendering their number non- statistically significant. TEM micrographs were acquired in a previous work on the model species *P. tricornutum* exposed to copper, and images include only 2–4 diatoms^22^. Another bottleneck related to the use of Electron Microscopy is the time needed for sample preparation, that requires sample fixation with toxic agents and several dehydration steps with alcohols. In light of this, we have opted for an experimental design that includes the estimation of cell concentration as broad indicator of a dose-dependent effect of toxicity of copper, and the FPM as image descriptor system. Our work followed a functional approach, i.e. we proposed to use an all-optical microscopy paradigm to characterize the effects HMs may produce on aquatic communities, which intrinsically depend on the concentration of their bioavailable fraction. In particular, we introduced a test that relies on three main concepts: i) The use of microalgae sensitive to the presence of HMs. ii) FPM as a multi-scale QPI method. iii) Multi-scale fractal analysis of FPM-QPI maps at the aim of assessing stress condition in the algal population when still capable of sustaining a positive growth. The use of FPM provides the unique opportunity to image the population and the liquid under test as a whole over a wide FoV, while returning super-resolved phase-contrast information at the single-cell level and in label-free mode.

Potential alternatives to the employed FPM apparatus within the QPI framework are in-line digital holography systems or different FPM configurations employing efficient source coding schemes. In-line digital holography by lensless configurations can ensure a quite wide FoV, i.e. as wide as the active area of the employed sensor^50^. However, resolution is inherently limited by the pixel pitch and it is strictly dependent on the adopted sensor. To keep the required resolution across the multiple scales and thus to permit the fractal analysis, we preferred to use FPM schemes. In our case, samples are fixed in lugol, so that time resolution was not a requirement. Whenever time resolution is important, or simply one wants to reduce the acquisition times for wide data collection campaigns, efficient source coding schemes implementing illumination multiplexing should be adopted^42,51–53^. Within the FPM framework, it has been demonstrated that the number of images can be reduced to 72 using annular bright-field illumination^52^ and up to 42 if one considers symmetrical dark-field illumination schemes^53^. This system improvement will be object of future investigation from our group.

We found that fractal analysis well manages the complexity of multi-scale FPM maps and can determine dose-dependent stress conditions in the microalgae populating the liquid under test. As a testbed for the proposed method, we use *Skeletonema Pseudocostatum* isolated from the Italian Sarno River mouth to probe the dose-dependent effects of the Cu bioavailable fraction. We conducted a study to investigate the optimal sample treatment protocol, (see Fig. 1A) and test modalities to optimize the sensitivity of the system to tiny dose variations that can provoke a non-negligible impact on diatoms. In these experiments, the initial cell density was relatively high, and algal cultures reached the late stationary phase within 72 h. This time frame is usually employed in several ecotoxicological assays aimed at assessing the effect of copper on diatoms and, in general, on model microalgal species^54–56^, allowing an immediate comparison between standardized and validated methods and the new protocol described in the present paper. We compared, in terms of system sensitivity, experiments conducted after exposing diatoms for 48 h and 72 h to different Cu doses, (i.e. T_2_ and T_3_). Although both conditions allowed us detecting the presence of Cu, we found that T_3_ is an optimal exposure to maximize the sensitivity. Instead, preliminary experiments performed at T_0_ and T_1_ showed poor system sensitivity, especially in the low and medium doses range, which means those exposure times are too short to obtain a non-negligible effect on this diatom species.

The proposed method is applicable in practice by sampling natural water from the location point under test, applying water filtering and/or digestion to remove the largest objects/particles, then to put *S. pseudocostatum* diatoms (from lab cultivation) and to let them in the liquid under test for the desired amount of time corresponding to ${T}_{3}$ for maximum sensitivity. In this way one could be certain about the time diatoms are exposed to Cu and can use them as bioprobes. In the conditions of our experiments, 72 h were sufficient to reach the late exponential phase in algal cultures. Additional experiments with less concentrated inocula of *S. pseudocostatum* could allow to evaluate the efficiency of this method to assess the effect of a longer-term exposure (7–10 days) of this species to Cu. Moreover, the efficiency on long timescales (e.g. 1 month) could be also assessed by employing other microalgal species – such as *Chlorophyceae* and *Eustigamtophyceae*—exhibiting slower growth rates (and thus longer growth curves) as test-organisms.

From the mere image analysis standpoint, results presented here are a first proof that multi-scale microscopes can reach their highest informative potential when coupled to analysis methods that consider the image features on multiple scales. Although this is a concept generalizable to a wider set of microscopy modalities and mathematical descriptors, we believe that fractal geometry is the most suitable candidate and can provide the optimal framework within this scope. It is worth to mention that FPM microscopes can be realized with non-demanding hardware components^31^ and in principle any benchtop optical microscope could be turned into a FPM setup by using a commercial LED matrix^28,30,31,42^. We believe this is an advantageous feature to broadly promote a new type of dose-dependent bioimpact-based ecotoxicological assays in biology labs.

### Supplementary Information

Supplementary Information.

## Data Availability

Documentation and analysis codes will be available upon reasonable request to the authors.
