# Supplementary material for: Multi-scale fractal Fourier Ptychographic microscopy to assess the dose-dependent impact of copper pollution on living diatoms
Source: Sci Rep. 2024 Apr 10;14:8418. doi: 10.1038/s41598-024-52184-3 (PMC11231145; doi:10.1038/s41598-024-52184-3)
Supplement: Supplementary file 1 — Supplementary Information. [file 41598_2024_52184_MOESM1_ESM.pdf]

# Supporting information for:

# Multi-scale fractal Fourier Ptychographic Microscopy to assess the dose-dependent impact of copper pollution on living diatoms

Vittorio Bianco,[1,§,*] Lisa Miccio,[1,§,*] Daniele Pirone,[1,§] Elena Cavalletti,[2] Jaromir Behal,[1,3] Pasquale Memmolo,[1] Angela Sardo,[2] Pietro Ferraro[1]

[1]*CNR-ISASI, Institute of Applied Sciences and Intelligent Systems "E. Caianiello", Via Campi Flegrei 34, 80078 Pozzuoli, Napoli, Italy*

[2]*Marine Biotechnology Department, Stazione Zoologica Anton Dohrn, Villa Comunale. 80121 Napoli, Italy.*

[3]*Department of Chemical, Materials and Production Engineering of the University of Naples Federico II, Piazzale Tecchio 80, 80125 Napoli, Italy*

§*These authors equally contributed to the present manuscript.*

**Corresponding authors: vittorio.bianco@isasi.cnr.it ; lisa.miccio@isasi.cnr.it*

## Preliminary operations: sampling, isolation, identification, exposure to Cu

*Skeletonema pseudocostatum* was isolated on June 2020 from water samples collected from the Sarno River mouth (40.7291N, 14.4698E). Specifically, water-sediment interface samples were collected in 100-mL sterile containers, and were stored at 4°C before processing. The species was initially maintained in small volumes (1 ml) in f/2 medium [20] at 30 salinity, amended with relatively high concentrations of Cu, Pb, Zn and Cd to test its resistance in HM-polluted environments. The species was then inoculated in 25-ml sterile flasks containing f/2 medium without further addition of HMs for ca. 2 months (to eliminate HM residuals), and adapted to a higher salinity, that is typical of marine environments. In particular, an aliquot of ca. 5 mL was poured in a Petri dish (diameter: 120 mm), diluted with the same volume of f/2 medium [20] (salinity 30) enriched with 5 mg $L^{-1}$ of $CuSO_4 \cdot 5H_2O$, 0.66 mg $L^{-1}$ of $Pb(NO_3)_2$, 14 mg $L^{-1}$ of $ZnSO_4 \cdot 7H_2O$, and 1.8 mg $L^{-1}$ of $CdCl_2$, and maintained for ca. 48 hours at low irradiance (ca. 50 µmol photons $m^{-2}s^{-1}$). A 1:1 dilution of natural seawater with modified f/2 lead to the following final molar concentrations:

10 µM of Cu, 1 µM of Pb, 25 µM of Zn and 5 µM of Cd. Heavy metal enrichment was used to promote the growth of those species able to survive in the presence of these persistent pollutants, and to space out living cells from each other. Isolation was performed under an inverted light microscope (Axiovert 200, Zeiss, Göttingen, Germany) at 200x magnitude with the capillary pipette method. Single cells were transferred in small (ca. 1 ml) sterile wells of a 24-multiwell plate containing f/2 medium amended with metals as described above [20] to stimulate the growth only in the case of resistance to HM pollution. The test-organism was initially maintained at low irradiance, and then transferred to 25-cm$^2$ sterile flasks containing f/2 medium prepared according to the classical recipe (e.g. without further addition of metals) at the beginning of the exponential phase. It was initially identified at genus level at optical microscope. To unequivocally identify the species, molecular identification of this diatom was performed on 50-mL samples by PCR amplification and sequencing of 18S and 28S genes. Before performing the experiments, *S. pseudocostatum* was adapted to a salinity of 36, in order to assess whether it could be used as indicator of heavy metal pollution in both brackish and marine environments. Small aliquots (1 ml) of the strain were weekly re-inoculated in a freshly prepared medium for ca. 2 months before performing the experiments. Cultures of *S. pseudocostatum* (initial cell density: 60000 cells/mL) were maintained in 75-cm$^2$ sterile flasks amended with f/2 medium (salinity 36) modified by dissolving different amounts of Cu sulfate pentahydrate (ACS Reagent grade, Sigma-Aldrich) to obtain the following final concentrations: 0 (e.g. control conditions), 5 µM, 10 µM, 25 µM, 35 µM and 50 µM. Each experiment was performed in triplicate. Diatoms were cultured for 72 h at 18°C under an irradiance of 160-180 µmol photons $m^{-2}$ $s^{-1}$ and a 12: 12 light: dark photoperiod. Their concentration was daily assessed by counting cells with a Bürker chamber. 5 mL of lugol-fixed samples were used to perform FPM analyses.

## FPM setup

The microscope objective (MO) was a 4x, Plan N, achromatic 0.1 NA from Olympus®. We used a 200 mm tube lens to transfer the image of the sample to a charge-coupled device (CCD) camera (Photometrics CoolSNAP Myo CCD) with 4.54 µm pixel pitch. We adopted a programmable LEDs matrix (Adafruit Industries) to generate probing beams from different directions. The sample was sequentially illuminated by each of the quasi-monochromatic red LEDs (632 nm wavelength, ~20 nm bandwidth) belonging to a circle with 60 mm radius, thus obtaining 177 low-resolution intensity images, each one sizing 1460×1940 pixels. The light source matrix was connected to an Arduino board, and the overall acquisition process was driven by a MATLAB® script. The overall optical system provided a 4.29×

magnification in the image plane. The low magnification permits accessing a wide 3.3 mm$^2$ FoV, while the FPM synthetic aperture principle allows super-resolved quantitative phase-contrast imaging. In particular, the setup adds a 0.5 illumination NA to the 0.1 NA of the MO. The resulting synthetic 0.6 NA corresponds to a 0.5 µm spatial resolution, and a large SBP = $6 \times 10^6$, which are far beyond the limits of the employed optical system. During the FPM reconstruction process, the entire FoV was divided into 14×19 patches of size 100×100, by considering 10 pixels overlap between neighbouring patches on each side. This choice is due to the need to fulfil the FPM assumptions and to optimize the computational efficiency [28]. The conventional FPM reconstruction algorithm is applied separately to each low-resolution 100×100 patch. After stitching all the 177 low-resolution intensities in the Fourier domain (as sketched in Fig. 1B), each patch gets converted into a high-resolution 500×500 complex amplitude, from which the sample amplitude and phase-contrast maps are obtainable. After stitching all the resulting patches, a 7000×9500 high-resolution PCM is obtained. Bright field and dark field images of a line target are shown along with the corresponding illumination in Fig. S1A. The retrieved HR amplitude Fourier spectrum in Fig. S1B is synthesized assuming contributions of all illuminating LEDs. In particular, frequency regions highlighted by the yellow circles are

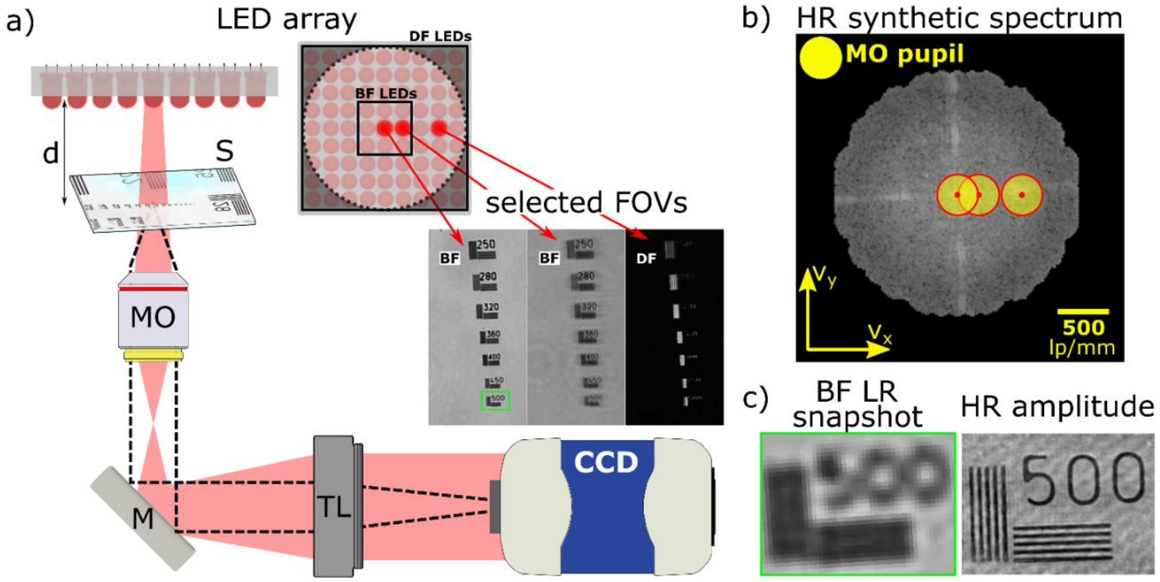


**Figure S1** FPM principle. A. Optical scheme of the FPM setup. S: studied object; d: distance between LED array and S; MO: microscope objective; M: mirror; TL: tube lens; CCD: camera. B: Synthesized amplitude Fourier spectrum. C: Bright-field low-resolution image and the corresponding high-resolution reconstruction.

determined by the used MO and their positions correspond to the switched-on LEDs in Fig. S1A. Finally, Fig. S1C provides comparison between the bright-field low-resolution image and the retrieved high-resolution amplitude. An example of recovered FPM-PCM of the samples is reported in Fig. 1B and Fig. 2 in the main text, which shows a very convenient trade-off between spatial resolution and FoV width.

## Fractal analysis

Each recorded PCM consists of 14×19 patches of size 500×500 square pixels, thus resulting in a high-resolution 7000×9500 image after stitching them.

1. As displayed in the $T_3$ control case in Fig. S2A (i.e., the case without Cu), we divided the PCM, $\psi$ , into 12 non-overlapping patches with size 2000×2000, namely fractal patches, $\psi_h$ , with $h = A, \dots, L$. At this aim, we cropped the overall PCM at 6000×8000 by cutting 500 pixels from the leftmost and the topmost areas, and 500 and 1000 pixels from the rightmost and the bottommost areas, respectively, as sketched in Fig. S2A.
2. Then, we normalized the cropped PCM between 0 and 1, and we padded each fractal patch with zeros until obtaining L=2048 pixels per side, see Fig. S2B. In fact, the numerical methods commonly employed to perform the fractal analysis in the discrete space usually require that the image under test has sizes equal to powers of 2 [41].
3. For each padded and normalized fractal patch, $\psi_h^{(P,N)}$, we created the hole maps $\Gamma_{(h,k)}$, with $k = 1, \dots, 4$, by binarizing them using thresholds that define the four intervals reported in Fig. S2C, i.e. 0≤φ≤0.25, 0.25<φ≤0.50, 0.50<φ≤0.75, and 0.75<φ≤1.
4. We carried out the lacunarity analysis for each of the hole maps $\Gamma_{(h,k)}$. Lacunarity is one of the most important fractal parameters. It can be defined as the distribution of the hole sizes and, as such, it requires a multi-scale analysis [44]. For this reason, the gliding box algorithm is commonly employed to compute lacunarity [41,46]. It consists in scanning the hole map at scale factors $\varepsilon = 2^0, 2^1, 2^2, \dots, 2^{\log_2(L)-1}$ , i.e. by gliding square boxes with sizes $r(\varepsilon) = L/\varepsilon$. For example, within the patch $\psi_A^{P,N}$ in Fig. S2B, the dashed white box with size $r = 512$ allows performing a gliding scanning at scale $\varepsilon = L/r = 4$.
    a. Let $S$ and $H(\varepsilon)$ be $L \times L$ and $r(\varepsilon) \times r(\varepsilon)$ matrixes of ones, respectively, and let $\Gamma$ be a generic hole map among the $\Gamma_{(h,k)}$ maps. The $A(\varepsilon)$ and $B(\varepsilon)$ images are computed as [41]

$$\begin{gathered} A(\varepsilon) = (1 - \Gamma) * H(\varepsilon) \\ B(\varepsilon) = S * H(\varepsilon) \end{gathered}, \tag{1}$$

where $*$ denotes the 2D convolution operator.

b. After dividing the frequency distribution of $A(\varepsilon)$ by the number of non-zero elements in $B(\varepsilon)$, we obtained the probability distribution $m(a, \varepsilon)$, with $a = 1,2, \dots, r^2$, and then we computed its first order moment, $p_1(\varepsilon)$, and second order moment, $p_2(\varepsilon)$, are computed.

c. Finally, at the scale ε, the lacunarity Λ can be defined as [41,46]

$$\Lambda(\varepsilon) = \frac{p_2(\varepsilon)}{p_1^2(\varepsilon)}. \tag{2}$$

d. Hence, lacunarity is not a single value, but a set of measurements able to describe the hole size distribution at the different scales denoted by the discrete variable ε. In Fig. S2D, the lacunarity curves $\Lambda_{h,k}(\varepsilon)$ are reported for each fractal patch $h = A, \dots, L$ and for each threshold $k = 1, \dots, 4$. Therefore, the lacunarity curve has an increasing trend with the scale factor ε and its values are large for small amounts of holes (as in case $\Lambda_{A,2}(\varepsilon)$ in Fig. S2D, corresponding to the hole map $\Gamma_{A,2}$ in Fig. S2C), while they approach the unity when the image is mostly made of gaps (e.g. $\Lambda_{A,1}(\varepsilon)$, $\Lambda_{A,3}(\varepsilon)$, and $\Lambda_{A,4}(\varepsilon)$ in Fig. S2D, corresponding to the hole maps $\Gamma_{A,1}$, $\Gamma_{A,3}$, and $\Gamma_{A,4}$ in Fig. S2C, respectively). In the extreme case of one single hole covering the entire image, $\Lambda(\varepsilon) = 1$ for any scale factor ε.

e. For each threshold $k = 1, \dots, 4$, the 12 lacunarity curves, $\Lambda_{h,k}(\varepsilon)$, are firstly averaged and then normalized between 0 and 1, thus obtaining the four curves $\bar{\Lambda}_k(\varepsilon)$, shown in Fig. S2E.

f. By using the trapezoidal rule, we calculated the area under curve (yellow regions in Fig. S2E) normalized to the maximum possible area (i.e., $\log_2(L) - 1$) as an ensemble descriptor of the lacunarity curves $\bar{\Lambda}_k(\varepsilon)$, namely $AUC_k$.

g. Finally, we defined the global lacunarity, GL, as the average value among the four $AUC_k$.

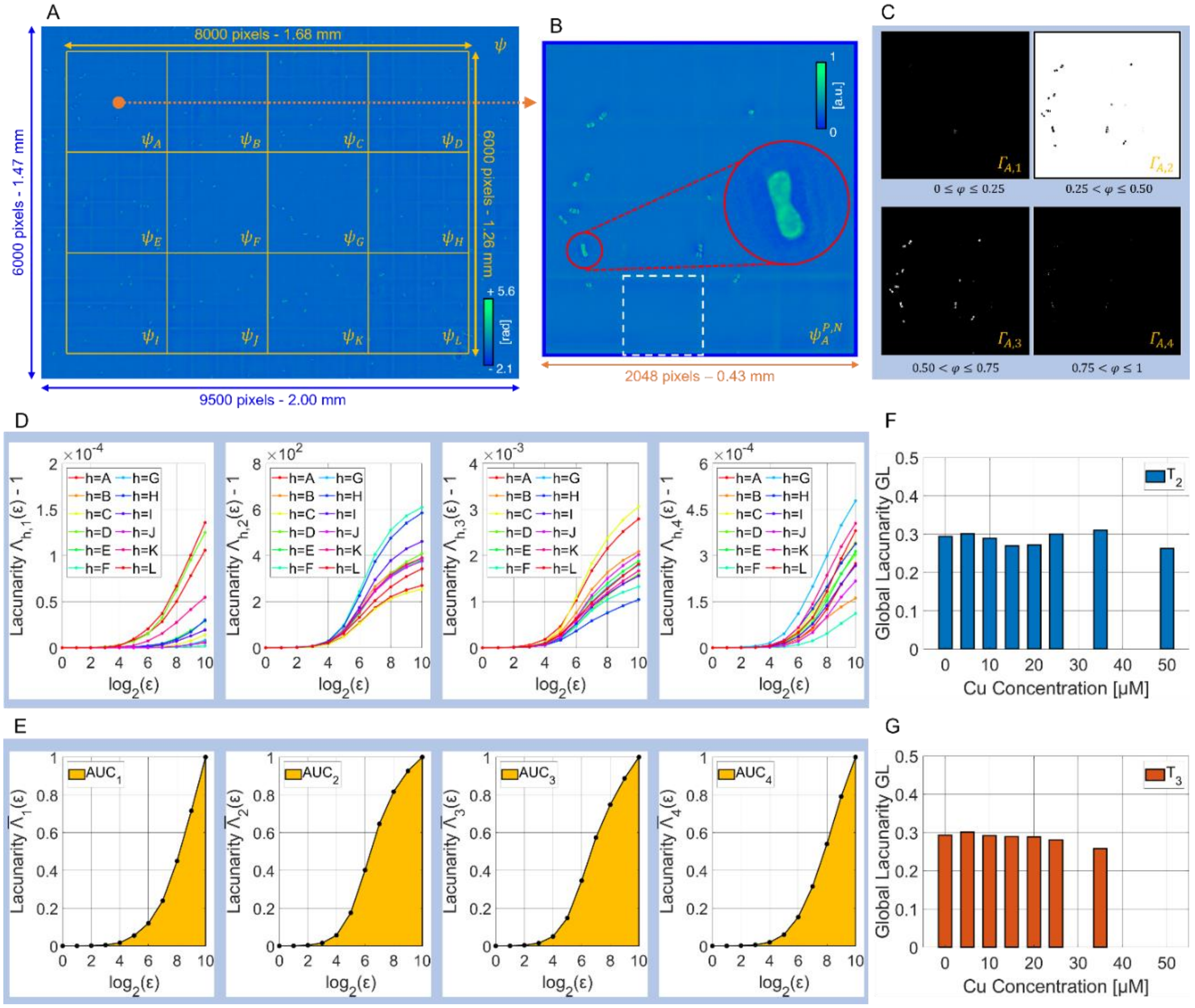


**Figure S2** Steps of the algorithm for computing the global lacunarity $\boldsymbol{GL}$, shown in the case of control diatoms without Cu doses at $T_3$ exposure time. A Full PCM with highlighted in yellow the fractal patches $\boldsymbol{\psi_h}$ with $\boldsymbol{h = A, \dots, L}$ within the cropped region used for the global lacunarity analysis. B Fractal patch $\boldsymbol{\psi_A}$ after zero-padding and normalization, with a diatom zoomed in the red inset. The square box with size $\boldsymbol{r = 512}$ used for the gliding scanning at the scale factor $\boldsymbol{\varepsilon = 4}$ is highlighted in white. C Hole maps $\boldsymbol{\Gamma_{A,k}}$ with $\boldsymbol{k = 1, \dots, 4}$ obtained after using the thresholds reported below. D Lacunarity curves $\boldsymbol{\Lambda_{h,k}(\varepsilon)}$ for each fractal patch $\boldsymbol{h = A, \dots, L}$ and for each threshold $\boldsymbol{k = 1, \dots, 4}$. E Averaged and normalized lacunarity curves $\boldsymbol{\overline{\Lambda}_k(\varepsilon)}$ with $\boldsymbol{k = 1, \dots, 4}$, with highlighted in yellow the area under curves $\boldsymbol{AUC_k}$. F,G Global Lacunarity $\boldsymbol{GL}$ at different Cu doses for the $T_2$ and $T_3$ exposure times, respectively, averaged among the three PCMs per dose.

Intrinsically connected to the way we defined and calculated it, GL takes into account the global context of the overall PCM, thus considering both the background medium and all the diatom probes inside it, while ranging from low to high scale factors, ε, and while spanning through the k threshold intervals. The GL curves at different Cu doses for the $T_2$ and $T_3$ exposure times are reported in Figs. S2F,G, respectively.

Instead, in order to investigate the sole single-diatom level, a local analysis has been performed.

1. At this aim, the 6000×8000 cropped PCM $\psi$ has been firstly low-pass filtered by a 25×25 average kernel, and then the gradient magnitude has been computed.
2. The resulting gradient PCM $\nabla\psi$ has been again divided into 12 fractal patches, $\nabla\psi_h$ (see Fig. S3A), as described at step 1 regarding global lacunarity.
3. After zero-padding each fractal patch at L×L (see padded fractal patch $\nabla\psi_A^P$ in Fig. S3B), the hole maps $\Gamma_{h,T}$ have been obtained by placing 0 to the pixels with $\nabla\varphi \geq T$ in order to segment diatoms from their background ($T = 0.15$), as displayed in Fig. S3C.
4. For each hole map $\Gamma_{h,T}$, the lacunarity curve $\Lambda_{h,T}(\varepsilon)$ has been computed (see Fig. S3D).
5. After averaging the 12 lacunarity curves $\Lambda_{h,T}(\varepsilon)$ and normalizing the resulting curve between 0 and 1, the average lacunarity curve $\bar{\Lambda}_T(\varepsilon)$ in Fig. S3E has been obtained.
6. Finally, we define the Local Lacunarity LL as the average lacunarity $\bar{\Lambda}_T(\varepsilon)$ at scale factor $\varepsilon = 64$, i.e. $\bar{\Lambda}_T(64)$ (yellow circle in Fig. S3E). In fact, the scale factor $\varepsilon = 64$ corresponds to the box size $r(64) = L/64 = 32$.

As shown in the inset in Fig. S3C, in our FPM imaging on average each diatom measures 128×128 square pixels, therefore the scale factor $\varepsilon = 64$ allows sensing the local changes at the single-diatom level ensuring that one element is covered by at least 4 boxes. The LL curves at different Cu doses for the $T_2$ and $T_3$ exposure times are reported in Figs. S3F,G, respectively. As shown in Figs. S2F and S3F, the global lacunarity GL and the local lacunarity LL are not monotonic with the Cu dose. For this reason, we define the multi-scale lacunarity as

$$MLS = 100\frac{GL}{LL} \qquad (5)$$

and we exploit it to measure the Cu concentration of a water sample.

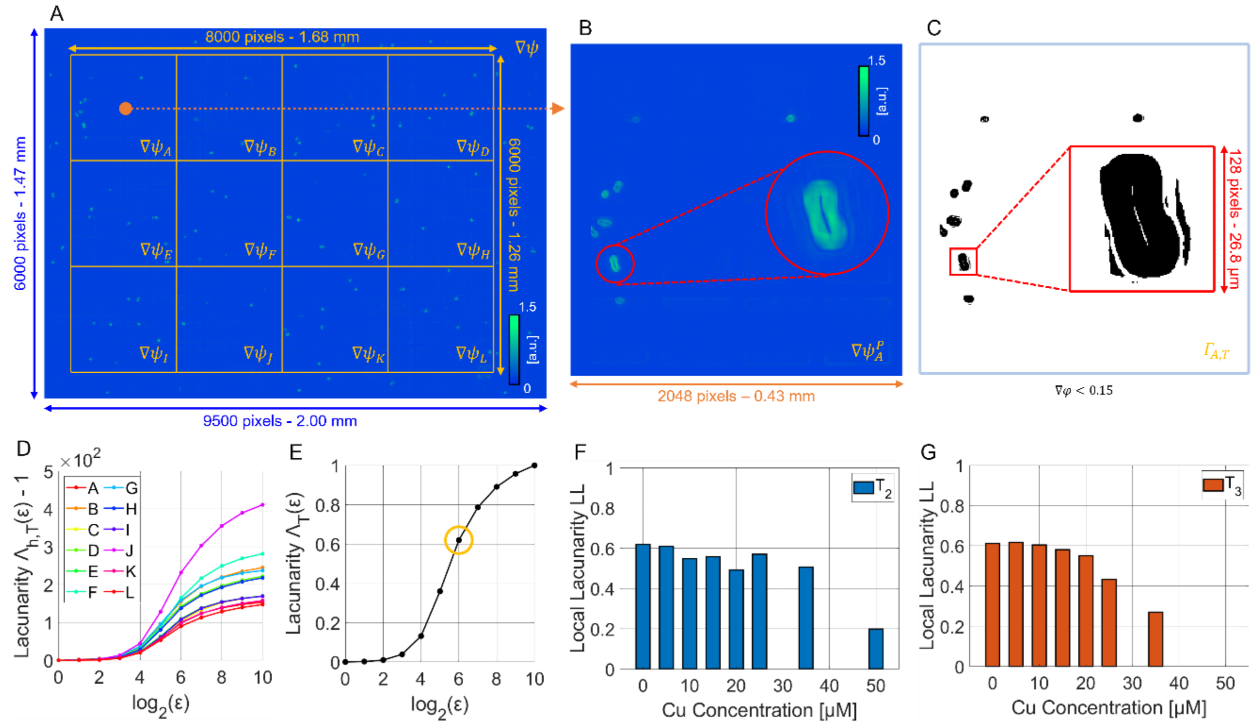


**Figure S3** Steps of the algorithm for computing the local lacunarity LL, shown in the case of control diatoms without Cu doses at $T_3$ exposure time. A Full gradient PCM $\boldsymbol{\nabla\psi}$ with highlighted in yellow the fractal patches $\boldsymbol{\nabla\psi_h}$ with h=A,…,L within the cropped region used for the local lacunarity analysis. B Fractal patch $\boldsymbol{\nabla\psi_A}$ after zero-padding, with a diatom zoomed in the red inset. C Hole map $\boldsymbol{\Gamma_{A,T}}$ obtained after using the threshold T reported below. D Lacunarity curves $\boldsymbol{\Lambda_{h,T}(\varepsilon)}$ for each fractal patch h=A,…,L. E Averaged and normalized lacunarity curve $\boldsymbol{\bar{\Lambda}_T(\varepsilon)}$, with highlighted in yellow the local lacunarity LL. F,G Local Lacunarity LL at different Cu doses for the $T_2$ and $T_3$ exposure times, respectively, averaged among the three PCMs per dose.

# Conventional features

In this section we compare the performance of the proposed fractal analysis approach to the results obtainable using some of the most common conventional features applied to the PCMs of the diatoms exposed to different Cu doses. At this aim, we considered the cropped PCMs divided into 12 fractal patches (Fig. S2A). For each $2000 \times 2000$ fractal patch, $\psi_h$, with $h = A, \dots, L$ (without applying zero-padding and normalization), the phase mean value, phase variance, and phase entropy were calculated and averaged among the patches, thus obtaining the $T_2$ and $T_3$ measurements at different Cu doses reported in Figs. S4A-C, respectively. Moreover, for each $2000 \times 2000$ fractal patch, $\psi_h^N$, with $h = A, \dots, L$ (after normalization but without applying zero-padding), we retrieved the binary mask, $M_h$, by selecting the values $\varphi^N < 0.25$ or $\varphi^N > 0.50$ values, since on average they can be related to the diatoms, as shown by the $\Gamma_{A,2}$ hole map in Fig. S2C (i.e., the complement of the binary mask $M_A$ without zero-padding). Then, for each $2000 \times 2000$ fractal patch, $\psi_h$, with $h = A, \dots, L$ (without zero-padding and normalization), we calculated the

phase mean value, phase variance, and phase entropy and we averaged them among the patches by only considering the pixels segmented in the corresponding binary masks, $M_h$, thus obtaining the $T_2$ and $T_3$ measurements at different Cu doses reported in Figs. S4D-F, respectively. In Fig. S4G, we display the percentage ratio between the area segmented in each binary mask $M_h$ and the overall patch area (i.e., $2000 \times 2000$) after averaging it across the patches $h = A, \ldots, L$ for each Cu dose. Furthermore, we calculated the Gray Level Co-occurrence Matrix (GLCM) in order to consider the different combinations of the gray levels found in the image. The GLCM $G(i, j, \theta, d)$ measures the number of times a pixel of value $i$ occurs at distance $d$ with respect to a pixel of value $j$ along the direction $\theta$. Therefore, a single image can have different GLCMs depending on the values of the offset $d$ and the angle $\theta$. Herein,

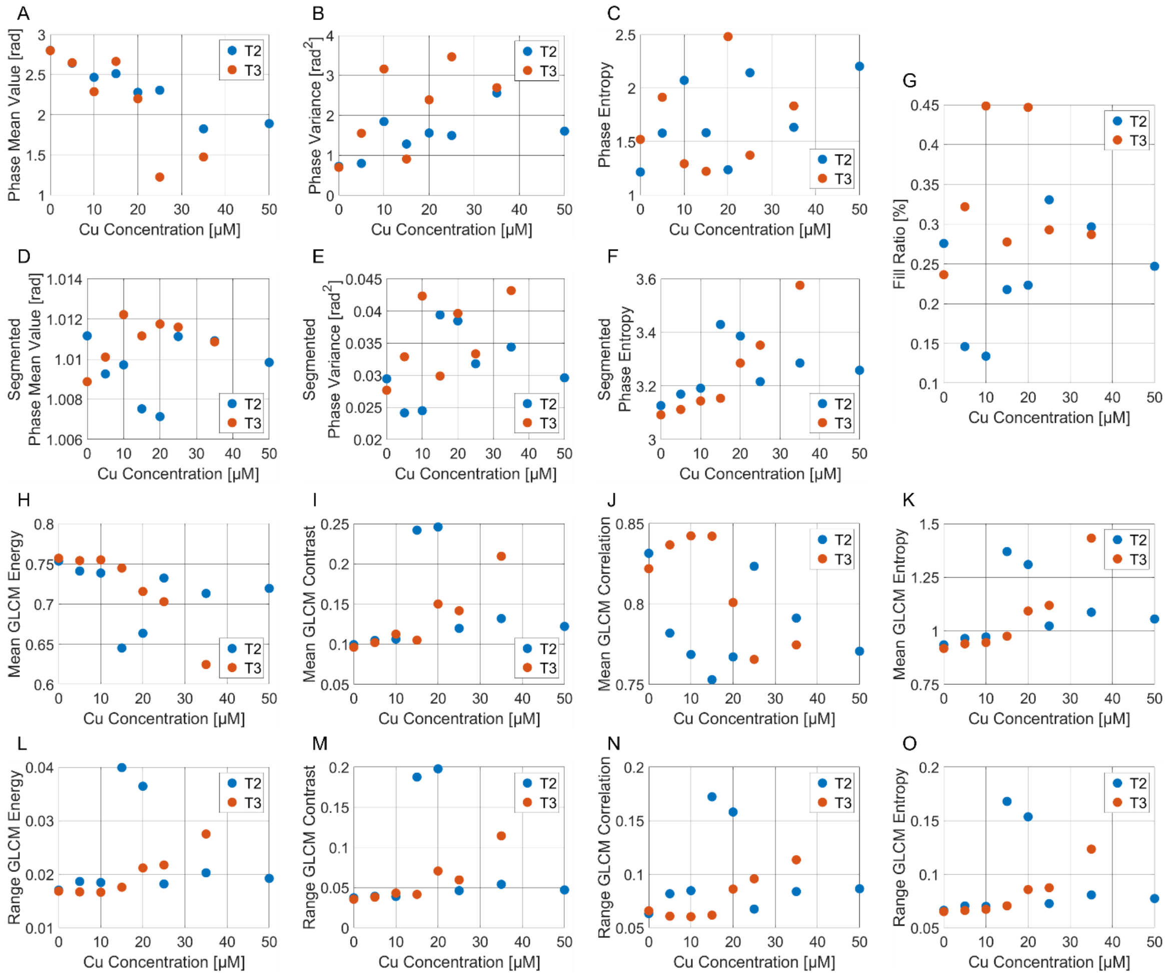


**Figure S4** Conventional features measured at different Cu doses after $T_2$ and $T_3$ exposure times. A-C Phase mean value, phase variance, and phase entropy, respectively, computed from the entire PCM. D-F Phase mean value, phase variance, and phase entropy, respectively, computed from the segmented PCM. G Percentage ratio between the area of the segmented PCM and the area of the overall PCM. H-O Mean values and ranges of the energy (H,L), contrast (I,M), correlation (J,N), and entropy (K,O), respectively, computed from the GLCMs.

for each $2000 \times 2000$ fractal patch, $\psi_h$, with $h = A, \dots, L$ (without zero-padding and normalization), four GLCMs have been computed at $d = 1$, i.e. $G_{h,\theta}$ with $h = A, \dots, L$ and $\theta = 0°, 45°, 90°, 135°$. For each GLCM $G_{h,\theta}$, we obtained the energy $e_{h,\theta}$, contrast $v_{h,\theta}$, correlation $c_{h,\theta}$, and entropy $s_{h,\theta}$ according to the Haralick definitions (S1). Then, for each $h = A, \dots, L$, we computed the average values $\bar{e}_h$, $\bar{v}_h$, $\bar{c}_h$, $\bar{s}_h$ and the ranges $\Delta e_h$, $\Delta v_h$, $\Delta c_h$, $\Delta s_h$ among the four energies $e_{h,\theta}$, contrasts $v_{h,\theta}$, correlations $c_{h,\theta}$, and entropies $s_{h,\theta}$ with $\theta = 0°, 45°, 90°, 135°$ (S2). Finally, we obtained the texture parameters by averaging across the patches $h = A, \dots, L$ the 12 mean values and ranges of the energy ($\bar{e}$ and $\Delta e$ in Figs. S4H,L, respectively), contrast ($\bar{v}$ and $\Delta v$ in Figs. S4I,M, respectively), correlation ($\bar{c}$ and $\Delta c$ in Figs. S4J,N, respectively), and entropy ($\bar{s}$ and $\Delta s$ in Figs. S4K,O, respectively), all of them calculated for each Cu dose. As a result of the comparison shown in Fig. S2, we selected the range GLCM entropy $\Delta s$ in Fig. S4O to improve the sensitivity of our system at the lowest Cu doses (i.e., the entropy-based texture parameter in Figs. 5C,D).

## SUPPLEMENTARY REFERENCES

[S1] R. M. Haralick, K. Shanmugam, and I. Dinstein, "Textural Features for Image Classification," Ieee Transactions Syst Man Cybern SMC-3, 610–621 (1973).

[S2] Costa, P. C., Guang, Z., Ledwig, P., Zhang, Z., Neill, S., Olson, J. J., & Robles, F. E. (2021). Towards in-vivo label-free detection of brain tumor margins with epi-illumination tomographic quantitative phase imaging. Biomedical optics express, 12(3), 1621-1634.
